# Medical students’ knowledge about COVID-19 and evaluation of the effectiveness of the applied preventive strategies

**DOI:** 10.1186/s13690-022-00873-8

**Published:** 2022-04-14

**Authors:** Kathie Sarzyńska, Eddie Czwojdziński, Amadeusz Kuźniarski, Sadri Rayad, Agnieszka Piwowar, Beata Jankowska-Polańska

**Affiliations:** 1 Department of Internal Medicine Nursing, Faculty of Nursing and Obstetrics, Wroclaw Medical University, 5 Bartla Street, 51-618 Wroclaw, Poland; 2 Department of Toxicology, Faculty of Pharmacy, Wroclaw Medical University, 50-556 Wroclaw, Poland; 3 Department of Prosthetic Dentistry, Faculty of Dentistry, Wroclaw Medical University, Wroclaw, Poland; 4 Research and Innovation Center, 4th Military Teaching Hospital, 50-981 Wroclaw, Poland

**Keywords:** Medical students, Knowledge, Education, Prevention, SARS-CoV-2, COVID-19

## Abstract

**Background:**

SARS-CoV-2 is a novel coronavirus which has caused a global pandemic. Due to the lack of available treatment for COVID-19 infections, prevention plays the most important role in combating the virus. Educational programs among students of medical faculties is necessary, because in the future they will act as health educators in the healthcare system. The aim of the study is to assess the students’ knowledge as an initial stage to the implementation of further preventive strategies against the spread of SARS-CoV-2 virus infections and to the evaluation of the effectiveness of the implemented preventive measures by continuous monitoring of the number of implemented administrative procedures.

**Methods:**

The study involved 482 students of medical faculties. Participants completed a questionnaire based on knowledge about the SARS-CoV-2 virus. Additionally, a number of preventive measures were introduced, including vaccination against COVID-19. During the entire period of the study, the number of administrative procedures (isolation and quarantine) were monitored.

**Results:**

The results of the knowledge test about COVID-19 were generally low and amounted to 11.0 (SD = 2.2). Significant differences in knowledge about COVID-19 between students of particular medical faculties were found. The most appropriate answers to questions about the incubation time of the SARS-CoV-2 virus were given by students of medicine (55%), followed by nursing (42.14%) and pharmacy (26%) students. Nursing students were the most correct in identifying the 3 main symptoms of COVID-19 (91.07%), followed by students of medicine (85.83%), and finally dentistry (77.27%) and pharmacy (76.67%) students. The Median (IQR) of students quarantined or isolating during steps 1, 2, 3, and 4 was 117,5 (142); 40 (43); 38, (20); and 9,5 (15), respectively.

**Conclusion:**

Students of certain faculties showed a low level of knowledge about transmission routes and procedures for dealing with a person suspected of being infected with SARS-CoV-2 or who has tested positive for COVID-19. Additional educational programs were conducted among medical students, along with other prevention strategies, which contributed to a decrease in the number of applied administrative procedures (isolation or quarantine).

## Background

COVID-19 is a new disease entity introduced by the WHO (World Health Organization) at the beginning of 2020. A new type of coronavirus called SARS-CoV-2 is responsible for its development. This virus shows high phylogenetic and clinical similarity to SARS-CoV but is characterized by higher transmission capacity and lower mortality [1]. Researchers have identified a number of symptoms associated with COVID-19 from the data collected. The most commonly observed symptoms are flu-like symptoms (fever, chills, fatigue and cough), cold symptoms (rhinitis, sneezing, sore throat and nasal congestion), joint and muscle pain, conjunctivitis, pulmonary symptoms (pneumonia, shortness of breath), gastrointestinal symptoms (including diarrhea, nausea), and loss of taste and smell or headache. Importantly, the course of COVID-19 may be multi-symptomatic or only slightly symptomatic. The SARS-CoV-2 virus is easily transmitted, mainly by droplets transferred from an infected person to a healthy person [2]. The death rate, estimated globally at 3.4% by the WHO, varies between countries and across ages [3]. However, this indicator is not constant and is changing due to the currently emerging new variants of SARS-CoV-2, which, as shown by new studies, in addition to greater infection capacity, increase the number of people hospitalized and, most importantly, may show higher mortality compared with the primary strain [4].

Currently, more and more complications after contracting COVID-19 are being observed. The most common neurological symptoms include headaches, dizziness, and disturbances in chemosensory functions (e.g., anosmia and ageusia). Reported symptoms also include significant mood swings and “brain fog”, which appear in some people even 2 to 3 months after the onset of the disease [5]. Numerous studies show that convalescents who have suffered a severe form of COVID-19 still require further therapy, especially pulmonary rehabilitation, aimed at improving exercise capacity [6–8].

The fight against the COVID-19 pandemic started in 2019 continues around the world to the present day. Currently used therapies are mainly based on alleviating symptoms, e.g. dyspnea, mainly through the supply of oxygen [9, 10]. In the face of an epidemiological threat, the governments of many countries decide to take measures to limit social contact (lockdown). The implemented strategies delay the development of the pandemic but are not able to prevent the occurrence of further SARS-CoV-2 virus infections. Due to this, preventive measures to ward off further transmission of the pathogen play a key role at present [11]. One such method is undoubtedly vaccination against COVID-19, the high effectiveness of which has been proven in clinical trials [12]. However, due to the voluntary nature of vaccination against SARS-CoV-2 and the concerns of part of the public regarding vaccination, the epidemiological situation is worsening in many countries. Poland is one of the countries with the lowest number of people willing to accept the COVID-19 vaccine (around 56.3%) [13]. Currently (01/2022) the estimates turned out to be overstated, as the % of persons fully vaccinated in Poland is 49.35 [14]. Due to the above, an additional method of prevention worth pursuing is focusing on educating the public on SARS-CoV-2 prevention [15].

Health education of the general society on COVID-19 (knowledge of prevention methods, transmission routes, epidemiological procedures) plays an important role in limiting the spread of the virus. The implemented methods of preventing SARS-CoV-2 virus infections should begin with educating a given group of people about the methods of transmission of the pathogen as well as the rules of conduct after close contact with a person who has tested positive for COVID-19 [16]. It is expected that as future specialists in health care services and disease prevention, students of medical faculties should demonstrate considerable knowledge in this area. Research shows that the state of knowledge about the SARS-CoV-2 virus among people working in other professions (not related to the medical industry) is lesser than that of medical professionals. Therefore, the aim of this study is to assess the level of knowledge about SARS-CoV-2 infection among students of various medical faculties at the Medical University in Wroclaw.

## Methods

**Fig. 1:**
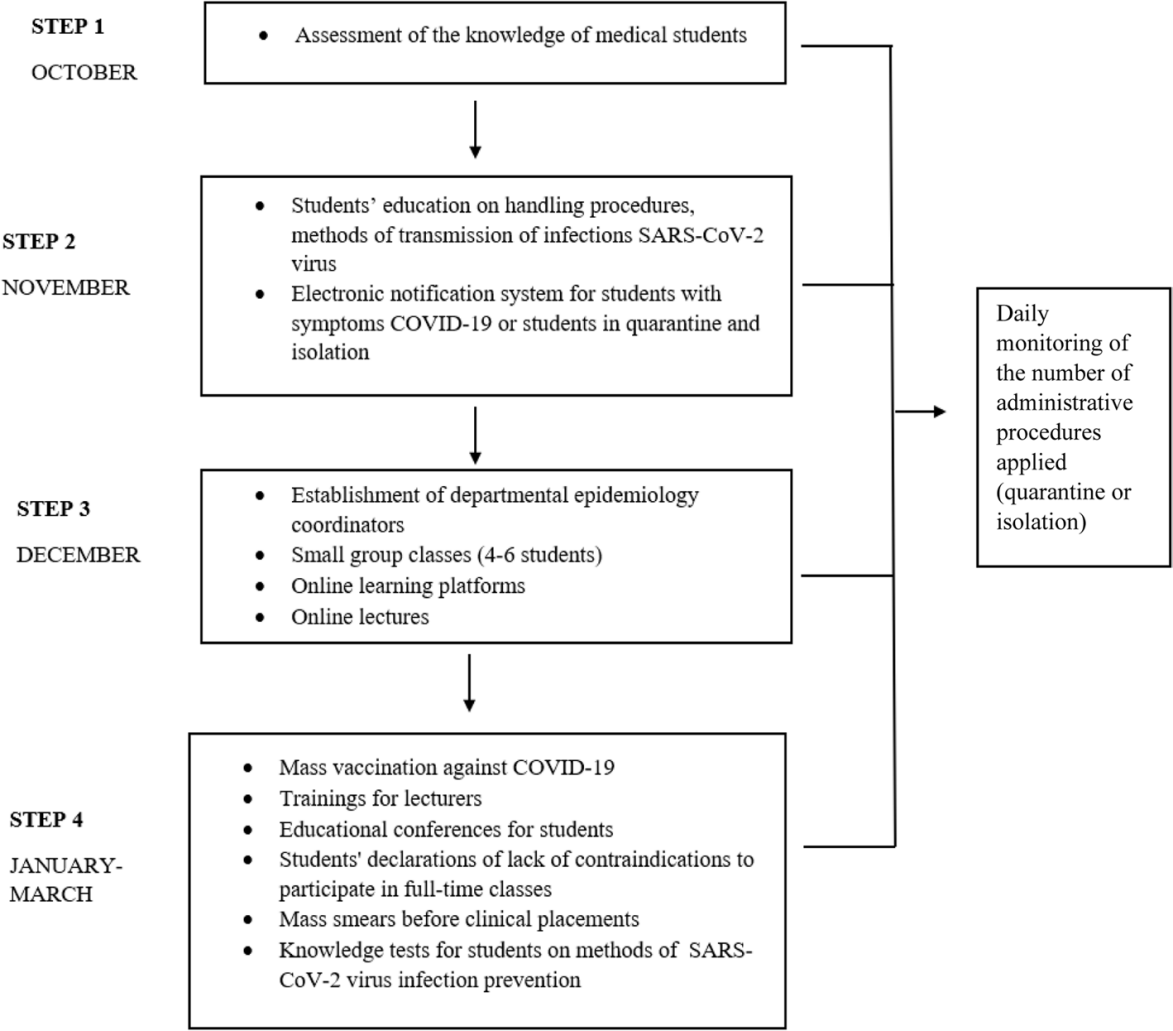
Study flow diagram

The study was conducted among students of several faculties of Wroclaw Medical University, i.e. nursing, medicine, dentistry and pharmacy, and lasted several months (from June 2020 to March 2021). The study was carried out in several successive stages (Fig. 1).

The study began with an assessment of students’ knowledge of prevention methods, SARS-CoV-2 virus transmission routes, and knowledge of how to proceed with a person suspected of being infected with SARS-CoV-2 or who has tested positive for COVID-19. The research utilized an original questionnaire, developed jointly by all the study co-authors, based on guidelines and information available on the WHO website, Polish government websites, and CDC (Centers for Disease Control and Prevention), and it was made up of two parts. The first 18 questions concerned general knowledge related to the SARS-CoV-2 virus. This part of the questionnaire contained 4 answers to choose from, of which only one was correct. Participants could earn 1 point for each correct answer. The maximum number of points was 18 (if 18 answers were correct). On the other hand, the second part of the survey consisted of 16 questions and concerned the verification of knowledge about the routes of transmission and prevention of the SARS-CoV-2 virus infection. In this part there were 3 answers to choose from: “I agree”, “I disagree”, “I do not know”, of which only one was correct. In accordance with survey design principles, comprehension of all questions was checked on a group of 20 individuals (pre-study) after the survey was created.

None of the respondents made any comments regarding the design of the questions or expressed any problems with understanding the survey. The next step was to use the created questionnaire to study the target population – medical students. The survey showed good psychometric properties. With Cronbach’s alpha coefficient greater than 0.8, the survey proved to be a good research tool. Data was gathered in an anonymized fashion.

Due to the epidemiological situation, corrective actions were necessary after conducting the research. They were adjusted for each of the departments in terms of the nature of the didactic course and took a form appropriate for the premises. Corrective actions consisted, inter alia, of educating students in the field of the new epidemiological threat and, above all, in the principles of proper use of personal protective equipment. A comprehensive educational program was developed, taking the form of an online lecture. A link to the lecture was sent to the students’ individual email accounts. The lecture was available for listening at any time.

The study was approved by the Wroclaw Medical University’s Bioethics Committee No. 617/2021.

### Statistical analysis

Statistical analysis was performed using the Statistica 13 program (TIBCO, Inc., USA). For measurable variables, arithmetic means and standard deviations were calculated. The frequency of occurrence (percentage) was calculated for qualitative variables. All investigated quantitative variables were checked with the Shapiro-Wilk test to determine the type of distribution. The comparison of qualitative variables between the groups was made using the chi-square test (χ2). Comparisons of the results were performed using the one-way analysis of variance (ANOVA) test and the post-hoc test (Tukey’s test) depending on the field of study. A significance level of α = 0.05 was assumed for all comparisons.

### Students

An invitation to participate in the study was sent by e-mail to students of 4 medical faculties (nursing, medicine, dentistry, pharmacy), using their individual university email addresses. Consent to participate in the study was conscious, and everyone who joined the survey confirmed their consent to informed, anonymous participation. In addition, participants clicked to confirm consent at the beginning of the questionnaire. Only after doing so could they begin solving it. No personal information was collected. The participants’ coded data were stored in a database. Each author of the paper had access to the coded database. Less than 40% of students (*n* = 502) responded to the invitation. A questionnaire was sent to the group of students who expressed their willingness to participate in the study. About 20 students were eliminated from the study because they did not specify their field of study. A total of 482 students were included in the study. The respondents were mostly women (83.2%), and the most numerous group were students of nursing (58.09%) (Table 1).

**Table 1:** Characteristics of the study group

Group *n* = 482			
		n	%
Gender	Female	401	83.2
	Male	81	16.8
Faculty	Medicine	120	24.90
	Nursing	280	58.09
	Dentistry	22	4.56
	Pharmacy	60	12.45

n – number of people; % - percentage

## Results

### Assessment of students’ knowledge on the topic of COVID-19

The overall results of the knowledge test about COVID-19 were low and amounted to 11.0 points (SD = 2.2) (Table 2). Students of selected medical faculties differed in their knowledge of the principles of prevention as well as that of SARS-CoV-2 virus transmission routes (see Tables 3 and 4).

**Table 2:** Results of the knowledge test

	Faculty								*P*-value	Total participants (*n* = 482)	
	Medicine		Nursing		Dentistry		Pharmacy				
	x̅	SD	x̅	SD	x̅	SD	x̅	SD		x̅	SD
Test results	11.16	2.1	11.14	2.3	10.14	2.4	10.75	2.0	0.129	11.0	2.2

**Table 3:** General knowledge about COVID-19

		Faculty								p
		Medicine		Nursing		Dentistry		Pharmacy		
		n	%	n	%	n	%	n	%	
**1. Select the three main symptoms of COVID-19:** Answer: fever, dry cough, dyspnoea	Right	103	85.83	255	91.07	17	77.27	46	76.67	**0.007**
	Wrong	17	14.17	25	8.93	5	22.73	14	23.33	
**2. What is the average incubation time of the virus:** Answer: 2–14 days	Right	66	55.00	118	42.14	10	45.45	16	26.67	**0.003**
	Wrong	54	45.00	162	57.86	12	54.55	44	73.33	
**3. What is the availability of drugs and treatment options:** Answer: No drugs are available	Right	24	20.00	61	21.79	8	36.36	17	28.33	0.257
	Wrong	96	80.00	219	78.21	14	63.64	43	71.67	
**4. What is the correct duration of disinfection:** Answer: 15–30 s	Right	81	67.50	219	78.21	10	45.45	44	73.33	**0.003**
	Wrong	39	32.50	61	21.79	12	54.55	16	26.67	
**5.The ideal alcohol strength for hand disinfection:** Answer: Above 60%	Right	99	82.50	242	86.43	18	81.82	55	91.67	0.368
	Wrong	21	17.50	38	13.57	4	18.18	5	8.33	
**6. Social distancing in preventing the transmission of infectious diseases means:** Answer: Non-pharmaceutical measures or measures taken to prevent the spread of an infectious disease by maintaining a physical distance of at least 2 m between people	Right	96	80.00	228	81.72	18	81.82	42	70.00	0.233
	Wrong	24	20.00	51	18.28	4	18.18	18	30.00	
**7. What does the term quarantine mean and to whom does it apply:** Answer: isolating a healthy person who was exposed to infection in order to prevent the spread of particularly dangerous and highly contagious diseases	Right	83	69.17	216	77.14	10	45.45	39	65.00	**0.004**
	Wrong	37	30.83	64	22.86	12	54.55	21	35.00	
**8. In case of exposure or contact with a source of disease caused by SARS-COV-2 (COVID-19) virus, apply:** Answer: Quarantine or epidemiological surveillance	Right	66	55.00	176	62.86	14	63.64	34	56.67	0.458
	Wrong	54	45.00	104	37.14	8	36.36	26	43.33	
**9. People who have been diagnosed with SARS-CoV-2 virus infection, contracted a disease caused by the SARS-CoV-2 (COVID-19) virus, or are suspected of infection or disease should be subjected to:** Answer: Mandatory isolation or isolation in domestic conditions	Right	49	40.83	105	37.50	6	27.27	16	26.67	0.221
	Wrong	71	59.17	175	62.50	16	72.73	44	73.33	
**10. After transferring a patient suspected of being infected with the SARS-CoV-2 coronavirus to an infectious diseases hospital:** Answer: all correct	Right	86	71.67	103	36.79	17	77.27	44	73.33	**< 0.001**
	Wrong	34	28.33	177	63.21	5	22.73	16	26.67	
**11. Staff who had close contact with a patient without the protection of appropriate personal protective equipment should:** Answer: be removed from work until the patient’s test result is known	Right	36	30.00	102	36.43	2	9.09	24	40.00	**0.035**
	Wrong	84	70.00	178	63.57	20	90.91	36	60.00	
**12. The definition of close contact with staff is:** Answer: all answers are correct	Right	93	77.50	232	82.86	16	72.73	41	68.33	0.061
	Wrong	27	22.50	48	17.14	6	27.27	19	31.67	
**13. Each patient presenting symptoms of an acute respiratory tract infection (fever > 38** °**C with cough or dyspnoea) in conjunction with the epidemiological criteria should be referred to the infectious or observational-infectious ward:** Answer: Yes	Right	39	32.50	146	52.33	8	36.36	21	35.00	**< 0.001**
	Wrong	81	67.50	133	47.67	14	63.64	39	65.00	
**14. How is the SARS-CoV-2 virus transmitted?** Answer: correct a and c (droplet and fecal-oral route)	Right	44	36.67	74	26.52	9	40.91	19	31.67	0.144
	Wrong	76	63.33	205	73.48	13	59.09	41	68.33	
**15. Among frequently used items, the greatest risk of transmission of SARS-CoV-2 is related to:** Answer: cell phones	Right	69	57.50	173	61.79	9	40.91	37	61.67	0.254
	Wrong	51	42.50	107	38.21	13	59.09	23	38.33	
**16. Contact Person – Low Risk Exposure is:** Answer: contact with the infected at a distance of> 2 m and time < 15 min. Longer contact increases the risk of transmission; The 15-min period of time is arbitrary. It may turn out that the degree of risk will be determined individually and action will be taken even in the case of a shorter duration of contact	Right	106	88.33	226	81.00	19	86.36	49	81.67	0.324
	Wrong	14	11.67	53	19.00	3	13.64	11	18.33	
**17. If an employee develops a fever, cough or shortness of breath during work, the basic correct action to be taken is:** Answer: immediate notification of the supervisor and the infection control team, withdrawal from work, identification of contact persons, isolation until swab test results are obtained, further follow-up depending on the result	Right	103	85.83	234	83.57	15	68.18	54	90.00	0.107
	Wrong	17	14.17	46	16.43	7	31.82	6	10.00	
**18. In case of infection, medical personnel under home isolation:** Answer: cannot leave the house due to isolation, should wait at home for the intervention team to come to collect a swab or follow the recommendations of the sanitary-epidemiological station	Right	96	80.00	236	84.29	17	77.27	47	78.33	0.531
	Wrong	24	20.00	44	15.71	5	22.73	13	21.67	

n – number of people; % - percentage

**Table 4:** Knowledge of SARS-CoV-2 prevention and transmission

		Faculty								p
		Medicine		Nursing		Dentistry		Pharmacy		
		n	%	n	%	n	%	n	%	
Maintaining personal hygiene and being socially responsible prevents the spread of COVID-19	I agree	120	100.00	278	99.28	22	100.00	60	100.00	0.963
	I disagree	0	0.00	1	0.36	0	0.00	0	0.00	
	I do not know	0	0.00	1	0.36	0	0.00	0	0.00	
The coronavirus is spread from person to person mainly by airborne droplets	I agree	120	100.00	276	98.57	22	100.00	60	100.00	0.820
	I disagree	0	0.00	3	1.07	0	0.00	0	0,00	
	I do not know	0	0.00	1	0.36	0	0.00	0	0,00	
Coronavirus can also spread through the ingestion of food	I agree	36	30.00	65	23.21	10	45.45	14	23.33	**0.001**
	I disagree	58	48.33	170	60.71	4	18.19	28	46.67	
	I do not know	26	21.67	45	16.08	8	36.36	18	30.00	
Washing your hands frequently with soap or disinfectant prevents the spread of COVID-19	I agree	119	99.17	276	98.57	22	100.00	60	100.00	0.952
	I disagree	1	0.83	3	1.07	0	0.00	0	0.00	
	I do not know	0	0.00	1	0.36	0	0.00	0	0.00	
Avoiding physical contact when greeting someone prevents the spread of COVID-19	I agree	115	95.83	272	97.14	22	100.00	59	98.33	0.866
	I disagree	2	1.67	4	1.43	0	0.00	1	1.67	
	I do not know	3	2.50	4	1.43	0	0.00	0	0.00	
Avoiding touching your eyes, nose, and mouth with your hands prevents the spread of COVID-19	I agree	118	98.33	276	98.58	22	100.00	59	98.33	0.715
	I disagree	2	1.67	2	0.71	0	0.00	0	0.00	
	I do not know	0	0.00	2	0.71	0	0.00	1	1.67	
Coughing and sneezing in your elbow or clothes is good practice for preventing the spread of COVID-19	I agree	100	83.33	255	91.08	20	90.91	53	88.33	0.255
	I disagree	11	9.17	16	5.71	0	0.00	4	6.67	
	I do not know	9	7.50	9	3.21	2	9.09	3	5.00	
Using a protective face mask when in contact with infected people protects against COVID-19 infection	I agree	58	48.33	200	71.43	11	50.00	28	46.67	**< 0.001**
	I disagree	50	41.67	69	24.64	7	31.82	27	45.00	
	I do not know	12	10.00	11	3.93	4	18.18	5	8.33	
Practicing social distancing and avoiding crowded places reduces the spread of COVID-19	I agree	120	100.00	278	99.29	22	100.00	60	100.00	0.694
	I disagree	0	0.00	0	0.00	0	0.00	0	0.00	
	I do not know	0	0.00	2	0.71	0	0.00	0	0.00	
For a person without COVID-19 symptoms, wearing a face mask is considered an appropriate and protective measure against COVID-19	I agree	65	54.17	209	74.65	16	72.72	35	58.34	**< 0.001**
	I disagree	39	32.50	58	20.71	3	13.64	23	38.33	
	I do not know	16	13.33	13	4.64	3	13.64	2	3.33	
Proper use of a face mask during a pandemic should include covering the nose, mouth and chin with the colored side facing outwards	I agree	114	95.00	262	93.57	22	100.00	55	91.67	0.417
	I disagree	2	1.67	13	4.64	0	0.00	2	3.33	
	I do not know	4	3.33	5	1.79	0	0.00	3	5.00	
Staying at home plays a vital role in preventing the spread of COVID-19	I agree	114	95.00	272	97.15	22	100.00	55	91.66	0.502
	I disagree	5	4.17	6	2.14	0	0.00	4	6.67	
	I do not know	1	0.83	2	0.71	0	0.00	1	1.67	
People with pre-existing, chronic diseases (heart disease, diabetes, high blood pressure and cancer, autoimmune diseases) are at higher risk of becoming infected with COVID-19	I agree	100	83.33	272	97.14	21	95.45	55	91.66	**< 0.001**
	I disagree	18	15.00	7	2.50	1	4.55	4	6.67	
	I do not know	2	1.67	1	0.36	0	0.00	1	1.67	
The elderly are the group most infected with COVID-19	I agree	29	24.17	116	41.43	10	45.45	11	18.33	**< 0.001**
	I disagree	83	69.16	158	56.43	11	50.00	45	75.00	
	I do not know	8	6.67	6	2.14	1	4.55	4	6.67	
Elderly people infected with COVID-19 are most often symptomatic	I agree	104	86.67	235	83.93	17	77.27	48	80.00	0.408
	I disagree	10	8.33	34	12.14	4	18.18	6	10.00	
	I do not know	6	5.00	11	3.93	1	4.55	6	10.00	
Young people are not infected with COVID-19	I agree	0	0.00	1	0.36	0	0.00	0	0.00	0.724
	I disagree	114	95.00	270	96.43	20	90.91	56	93.33	
	I do not know	6	5.00	9	3.21	2	9.09	4	6.67	

n – number of people; % - percentage

There were no significant differences in the level of general knowledge between students of the 4 selected fields of study. The obtained results were the highest for the faculty of medicine at 11.16 points (SD = 2.1) and the lowest for the faculty of dentistry at 10.14 points. (SD = 2.4) (Table 2).

One of the elements of the study was to assess the level of knowledge about COVID-19 among students from individual years of education (from 1 to 6). In the overall results of the COVID-19 knowledge test, no significant statistical differences were found (*P* = 0.129).

However, first year students gave slightly less accurate answers compared to students in later stages of education. Significant differences concerned the following issues (*P* < 0.05): the rules for proceeding with a patient after transporting them to an infectious disease hospital (46.89% of first year students answered incorrectly, while only 22.22% fifth year students were incorrect), further treatment of people who tested positive for SARS-CoV-2, routes of coronavirus transmission as well as determining whether the elderly are more susceptible to SARS-CoV-2 infection (students of the faculty of medicine gave as many as 41.24% incorrect answers).

Among students of all faculties, knowledge gaps were found regarding issues such as (Table 3):

- Duration of hand disinfection (15–30 s) – nursing students showed the highest level of knowledge in this aspect (78.21%), followed by pharmacy students (73.33%), medicine students (67.5%) and finally dentistry students (45.45%).
- Identification of the 3 main symptoms of COVID-19, i.e. fever, dry cough, and shortness of breath. Nursing students (91.07%) gave the most accurate answers, medical students (85.83%) did a little worse, while students of dentistry (77.27%) and pharmacy (76.67%) scored the least points.
- Procedures for further treatment of a person suspected of being infected with SARS-CoV-2 in an infectious disease hospital. The correct procedure was best known to students of dentistry (77.27%), followed by pharmacy students (73.33%). Students of medicine (71.67%) gave slightly less correct answers, while nursing students (36.79%) showed the least knowledge on the topic.
- Duration of SARS-CoV-2 incubation (2–14 days). Only a little more than every fourth pharmacy student (26%) knew the incubation time of the SARS-CoV-2 virus, which is 2–14 days. Nursing students (42.14%) gave slightly more correct answers to the above question, and the highest results were obtained by medicine students – 55%.
- Dealing with personnel who have had close contact with a patient suspected of being infected with SARS-CoV-2 without the protection of appropriate personal protective equipment. Most of the students participating in the study did not know that in such a situation staff should be removed from work until the suspected patient’s results have been brought back. Students of pharmacy (40%), followed by nursing (36.43%) and medicine (30%), showed the most knowledge in this respect. Only every tenth student of dentistry gave a correct answer to the above question (9.09%).
- The meaning of the word quarantine (isolating a healthy person who has been exposed to a pathogen in order to prevent the spread of particularly dangerous and highly infectious diseases). Most of the nursing students (77.14%) gave the correct answer to the above question. Students of medicine (69.17%) and pharmacy (65%) fared slightly worse. Students of dentistry showed the greatest deficit when it came to the correct definition of quarantine, with less than half of the students knowing the correct answer.
- Principles of managing a patient presenting symptoms of acute respiratory infection (fever > 38 °C with cough or dyspnea) in accordance with epidemiological criteria (the patient should be sent to the infectious or observational-infectious ward). More than half of the nursing students (52.33%) knew the correct answer to the above question. Knowledge gaps were easier to notice in students of faculties of medicine, dentistry and pharmacy, where the percentage of correct answers was as follows: 32.5, 36.36, 35%.

In the second part of the survey, students declared whether or not they agreed with a set of statements about COVID-19 (Table 4). Unfortunately, the students did not know that the SARS-CoV-2 virus can also be transmitted via the oral route (the largest knowledge deficit in this matter was presented by nursing students, with as many as 60.71% incorrect answers). Moreover, students had difficulty choosing whether the use of a protective face mask when in contact with infected people protects against infection with the SARS-CoV-2 virus (the most inaccurate answers were given by pharmacy students, with 45% incorrect answers). Determining whether the elderly are more likely to become infected with COVID-19 was another problematic issue for study participants (the most incorrect answers were given by students of dentistry faculty – 45.45% were incorrect).

### Monitoring and evaluation of applied administrative procedures (isolation and quarantine)

**Fig. 2:**
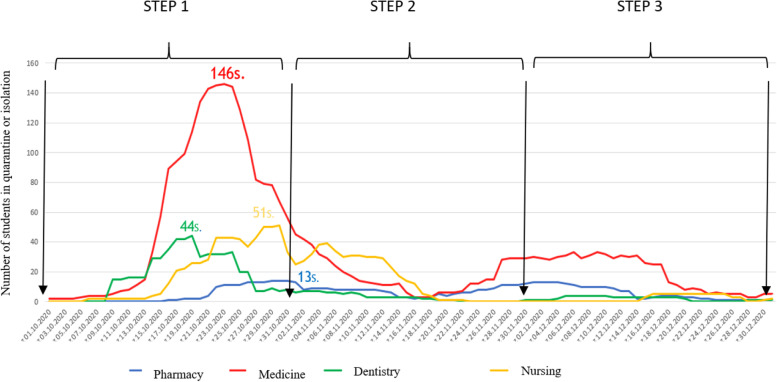
Number of administrative procedures applied to medical students of each faculty (pharmacy, medicine, dentistry and nursing) in 2020, months of X-XII. Step 1: assessment of the knowledge of medical students; Step 2: students’ education on handling procedures, methods of transmission of infection with the SARS-CoV-2 virus, electronic notification system for students with symptoms COVID-19 or students in quarantine and isolation; Step 3: faculty epidemiology coordinators, small group classes (4–6 students), online learning platforms, online lectures

**Fig. 3:**
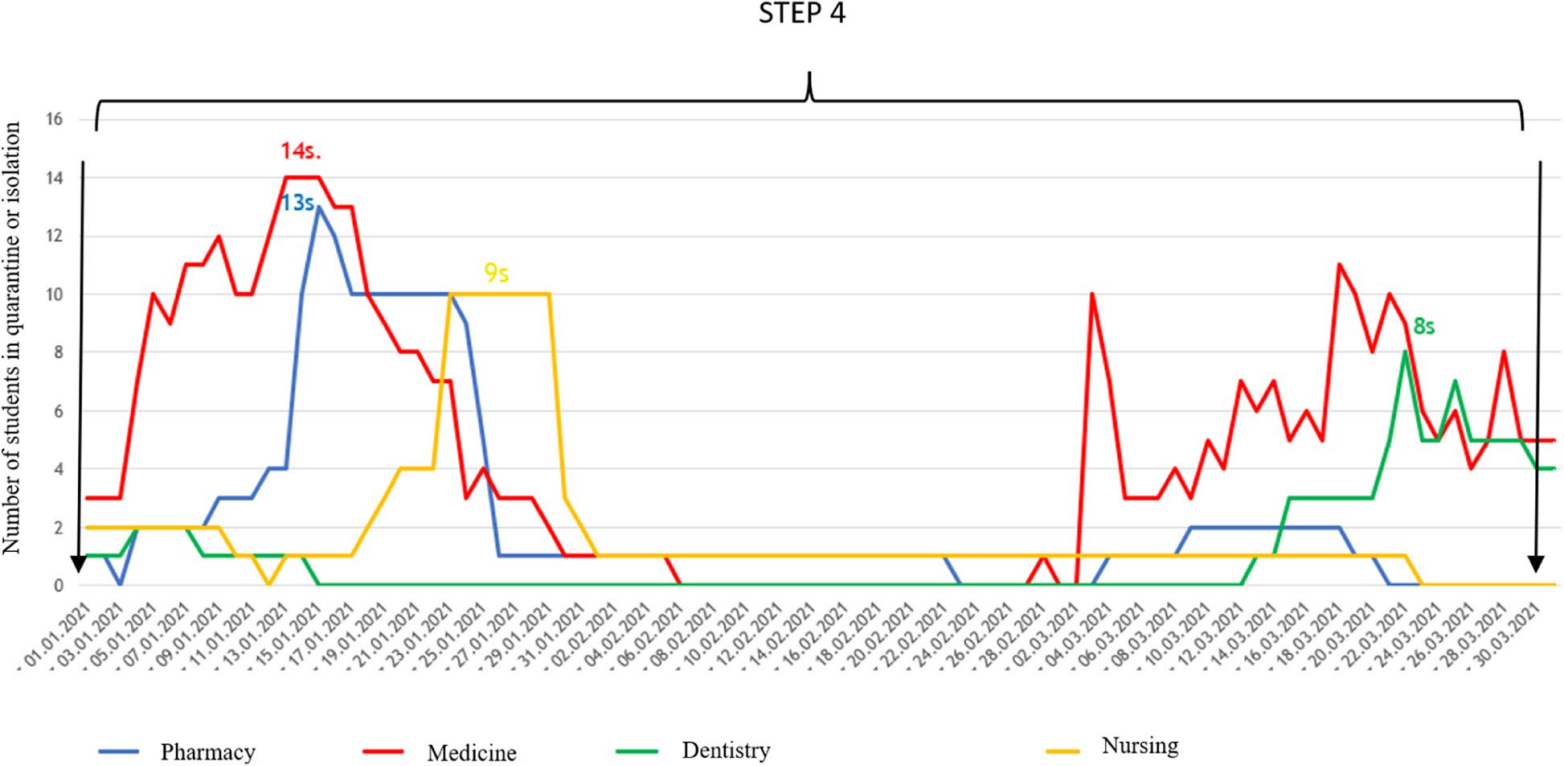
Number of administrative procedures applied to medical students of each faculty (pharmacy, medicine, dentistry and nursing) in 2021, months I-III. Step 4: mass vaccination against COVID-19, trainings for lecturers, educational conferences for students, students’ declarations of lack of contraindications to participate in full-time classes, mass smears before clinical placements, knowledge tests for students on methods of SARS-CoV-2 virus infection prevention

A comparison of the number of applied administrative procedures (quarantine or isolation) among students of the 4 studied faculties was made. The study focused on the time period between October 2020 and March 2021, dividing it into two stages: the first from October 2020 to December 2020 (steps 1, 2 and 3), as this was the period before the commencement of mass vaccination in group 0 (medical field professionals and medical students) (Fig. 2). The second period lasted from January 2021 to March 2021, covering mass vaccination of students against COVID-19 (Fig. 3). In both periods of the study, it can be observed that students of the faculty of medicine underwent the highest number of administrative procedures – quarantines and periods of isolation. A particularly high number was observed in the period from October 9, 2020 to November 2, 2020, compared to students of other medical studies (Fig. 2). It should be noted that there was a significant difference in the maximum values ​​of the number of people staying in isolation and quarantine on a given day between individual fields of study: Medicine – 146 students; Health Sciences – 51 students; Dentistry – 44 students and Pharmacy – 13 students. In the same period of time, an increase in the number of administrative procedures in other fields of study was be observed compared to the later period, i.e. after 2nd November 2020. The month of October 2020 was devoted to educating students on prevention methods, routes of transmitting the SARS-CoV-2 virus and procedures that should be applied after coming in contact with a person who has tested positive for SARS-CoV-2.

Mass vaccination in the departments in January 2021 was an important factor that may have limited the spread of the virus. The measures taken resulted in a significant reduction in the number of quarantines and periods of isolation among students of all faculties. The largest decrease can be observed in the case of the Faculty of Medicine, with the highest number of per day administrative procedures in the first period (from October 1st to January 1st) – 144 subjected students (Fig. 2) – and zero daily administrative procedures in the second period of the study (Fig. 3).

The daily number of new administrative procedures was greatest at stage 1, when only the assessment of students’ knowledge was carried out as the starting point for the development of further prevention strategies. The study showed that educating students about the SARS-CoV-2 virus contributed to a decrease in the number of daily epidemiological procedures used (isolation or quarantine). The analysis of the applied steps made it possible to conclude that the successive introduction of preventive measures allowed to maintain a downward trend in the number of daily epidemiological procedures used.

During step 1, the mean number of daily isolation or quarantine cases was up to 10 times greater than during step 4, where the key point was undoubtedly the introduction of COVID-19 vaccines. It allowed for a significant reduction in the curve of infection and a further downward trend (less quarantines and periods of isolation). The median (IQR) of students quarantined or isolated during steps 1, 2, 3, and 4 was 117,5 (142); 40 (43); 38 (20); and 9,5 (15), respectively. The greatest decrease was seen in step 2, when the process of educating students about prevention methods and ways of spreading the SARS-CoV-2 virus began (52,9% less cases compared to step 1) and after the initiation of mass student vaccination in step 4 (73,7% less cases compared to step 3).

In conclusion, a number of measures were taken to prevent medical students from contracting the SARS-CoV-2 virus. The goal was to reduce the transmission of the SARS-CoV-2 virus among university students. In the initial period of the study, the greatest intensification of activities was related to increased emphasis on the effectiveness of educational programs. In addition to education, a number of the above-mentioned additional activities were carried out to ensure the safety of students and to take quick action. These included continuous monitoring of the number of infections and high-risk contacts combined with an analysis of epidemic outbreaks by a group of specialists – a crisis team formed at the beginning of the first wave of the pandemic, i.e. in March 2020. As a result of the actions taken, a decrease in the number of applied administrative procedures (isolation or quarantine) was observed. The downward trend continued throughout the study. Considering the above, it is worth noting that the factors that showed the best preventive measures against SARS-CoV-2 infection in the conducted study were education and mass vaccination against COVID-19.

## Discussion

The study of the knowledge of medical students on transmission routes, prevention and rules of conduct in relation to a person suspected of being infected with the SARS-CoV-2 virus or who tested positive for COVID-19 is the first study of this kind to be conducted in Poland. Medical students are a group that supports infectious disease hospitals in the fight against the coronavirus pandemic by assisting in the provision of healthcare services and many other activities much needed during a health crisis on an unprecedented scale [17]. One of the main conditions of safe work is knowledge of the principles of prevention and epidemiological procedures as well as compliance with said procedures [18]. Gaps in knowledge may contribute to easy transmission of the SARS-CoV-2 virus. Although the main concern in a pandemic is to provide health care to reduce mortality, learning how to avoid the infection in the first place plays an important role.

A comparative analysis of the students’ level of knowledge on the principles of prevention and the routes of SARS-CoV-2 virus transmission has not been published so far in the same form as our study. Researchers present the knowledge of medical students about COVID-19 as a collective group with no comparison between fields of study. In assessing the state of knowledge about COVID-19, researchers often use self-designed questionnaires, which creates difficulties in comparing the knowledge of medical students with other studies conducted around the world due to the different meanings of the words “good” or “bad” knowledge [19].

The overall result of the COVID-19 knowledge test employed in the study was 11.0 (SD = 2.2), which can be interpreted as low. A similar study with the use of the same research tool was conducted on a group (*n* = 185) of medical employees of 3 care and treatment centers, located in Legnica (approximately 70 km from Wroclaw). The results of the general knowledge test about COVID-19 were slightly lower, and were as follows for individual facilities: 8.89 (SD = 2.5) vs 10.48 (SD = 3.3) vs 10.08 (SD = 2.4) [20]. This may prove that there is a deficit of knowledge about COVID-19 not only in the studied group of medical students but also among people who actively provide healthcare services to patients. The level of knowledge about COVID-19 will presumably be even lower for people who do not have a medical education, but so far research on such a group has not been conducted in Poland.

One of the significant knowledge gaps was related to ignorance of the SARS-CoV-2 virus incubation time. Over one in four pharmacy students (26%) were able to correctly indicate the aforementioned incubation time as 2–14 days. Nursing students gave a slightly higher number of correct answers to the above question (42.14%), and the best results were obtained by students of medicine – 55%. Medical students have also shown greater knowledge in this regard in other studies, with as many as 84% correct answers [21]. In turn, in the work of Hasan et al. [22], 69% medical students from the United Arab Emirates were able to indicate the incubation time of SARS-CoV-2 as less than 14 days. However, in another one of our studies in Poland, health care professionals provided only 52 and 64% correct answers regarding this subject [20]. Doctors have shown even less knowledge of the incubation time of SARS-CoV-2 – only 45.3% correct answers [23]. The lack of knowledge about the incubation time of SARS-CoV-2 among medical students seems to be worrying, because it is knowledge of the incubation time of the virus that determines the planning of epidemiological treatment for a patient who has been in contact with a person with a confirmed positive COVID-19 test result.

Additionally, in our study, it was shown that the vast majority of students knew the 3 main symptoms of COVID-19, i.e. fever, dry cough and shortness of breath. Nursing students were the most accurate, with 91.07% correct answers; medicine students (85.83%) did a bit worse; and students of dentistry (77.27%) and pharmacy (76.67%) demonstrated the most noticeable knowledge gaps. In another study, more than 92% of medical students from United Arab Emirates were able to identify these 3 main symptoms as the most commonly associated with COVID-19 infection [22]. The study did not show a correlation between the duration of studies and the level of general knowledge of the studied groups about the SARS-CoV-2 virus. This may be due to the fact that COVID-19 is a new disease entity, and knowledge deficits regarding the subject may affect students of any year. On the other hand, research conducted among medical students in Pakistan showed that students of the last (fifth) year knew more about COVID-19 compared to students at an earlier stage of education – 61.1% correct answers among first year students vs. 82.3% among fifth year students [24]. In another study conducted in Turkey among medical students in their final year of study, the level of knowledge was defined as average. However, researchers did not include lower-year students in the study [25].

The differences between our findings and that of other authors may be related to the information chaos that was present at the beginning of the pandemic. At the time of study (June 2020), there was a lot of false information about the routes of transmission and prevention of the SARS-CoV-2 virus infection, even coming from people closely related to the medical industry. It was common to promote knowledge that at that time was not supported by scientific research due to the lack of publications in the field. It is well known that knowledge gaps directly increase the risk of transmission of pathogens and can thus create new infection outbreaks. It is recommended to disseminate knowledge on counteracting COVID-19 among other social groups, as carrying out such activities can effectively prevent new cases of infections and reduce the transmission of coronavirus. Future healthcare professionals play a key role in disseminating correct and factual messages to the public. In addition, they also act as role models for society. Therefore, it is imperative for future healthcare professionals to rely on a correct up-to-date COVID-19 knowledge base [21]. Currently, the best strategy to prevent SARS-CoV-2 transmission seems to be education (training) and the popularization of COVID-19 vaccination.

## Conclusions

Students of certain faculties showed a low level of knowledge regarding transmission routes and procedures for dealing with a person suspected of being infected with the SARS-CoV-2 virus or with a positive COVID-19 result. Determining the knowledge deficits of medical students is the basis for developing educational programs aimed at minimizing the detected gaps. Educational courses conducted among medical students contributed to a decrease in the number of epidemiological procedures (isolation or quarantine) at the university. Health education and compliance with epidemiological recommendations, along with the vaccination process, seem to currently be the best methods of prevention, with a real impact on reducing the number of SARS-CoV-2 virus infections.

### Study limitations

The study had its limitations. Due to the lack of available standardized tools to measure knowledge about the prevention and spread of SARS-CoV-2 at the time of the survey, an original questionnaire was used. The questionnaire for our study was developed based on information available on WHO, CDC, and Polish government websites. Another limitation was the inclusion of medical students from only one research center in the study. The knowledge of medical students was assessed only in 4 selected medical faculties. Obstetrics, physiotherapy, medical emergency and public health students did not participate in the study.

The study did not reassess students’ knowledge, as the main objective was to assess the level of students’ knowledge of SARS-CoV-2 virus transmission routes to identify students’ knowledge gaps in this aspect and adapt educational programs to minimize these gaps.

## Data Availability

All data is available from the correspondent author.

## Abbreviations

WHO: Word Health Organization
CDC: Centers for Disease Control and Prevention
